# Advancing Kiwifruit Maturity Assessment: A Comparative Study of Non-Destructive Spectral Techniques and Predictive Models

**DOI:** 10.3390/foods14152581

**Published:** 2025-07-23

**Authors:** Michela Palumbo, Bernardo Pace, Antonia Corvino, Francesco Serio, Federico Carotenuto, Alice Cavaliere, Andrea Genangeli, Maria Cefola, Beniamino Gioli

**Affiliations:** 1 Institute of Sciences of Food Production, National Research Council of Italy (CNR), c/o CS-DAT, Via M. Protano, 71121 Foggia, Italy; michelapalumbo@cnr.it (M.P.); antonia.corvino@ispa.cnr.it (A.C.); 2 Institute of Sciences of Food Production, National Research Council of Italy (CNR), Via G. Amendola, 122/O, 70126 Bari, Italy; francesco.serio@cnr.it; 3 Institute of Bio Economy, National Research Council of Italy (CNR), Via Gobetti 101, 40129 Bologna, Italy; federico.carotenuto@ibe.cnr.it; 4 Institute of Bio Economy, National Research Council of Italy (CNR), Via Caproni 8, 50145 Firenze, Italy; alice.cavaliere@cnr.it (A.C.); a.genangeli@gmail.com (A.G.); beniamino.gioli@cnr.it (B.G.); 5 Institute of Polar Sciences National Research Council of Italy (CNR), Via Gobetti 101, 40129 Bologna, Italy

**Keywords:** *Actinidia chinensis* L., maturity index, glucose, fructose, soluble solids content, Gaussian process regression, hyperspectral analysis

## Abstract

Gold kiwifruits from two different farms, harvested at different times, were analysed using both non-destructive and destructive methods. A computer vision system (CVS) and a portable spectroradiometer were used to perform non-destructive measurements of firmness, titratable acidity, pH, soluble solids content, dry matter, and soluble sugars (glucose and fructose), with the goal of building predictive models for the maturity index. Hyperspectral data from the visible–near-infrared (VIS–NIR) and short-wave infrared (SWIR) ranges, collected via the spectroradiometer, along with colour features extracted by the CVS, were used as predictors. Three different regression methods—Partial Least Squares (PLS), Support Vector Regression (SVR), and Gaussian process regression (GPR)—were tested to assess their predictive accuracy. The results revealed a significant increase in sugar content across the different harvesting times in the season. Regardless of the regression method used, the CVS was not able to distinguish among the different harvests, since no significant skin colour changes were measured. Instead, hyperspectral measurements from the near-infrared (NIR) region and the initial part of the SWIR region proved useful in predicting soluble solids content, glucose, and fructose. The models built using these spectral regions achieved R^2^ average values between 0.55 and 0.60. Among the different regression models, the GPR-based model showed the best performance in predicting kiwifruit soluble solids content, glucose, and fructose. In conclusion, for the first time, the effectiveness of a fully portable spectroradiometer measuring surface reflectance until the full SWIR range for the rapid, contactless, and non-destructive estimation of the maturity index of kiwifruits was reported. The versatility of the portable spectroradiometer may allow for field applications that accurately identify the most suitable moment to carry out the harvesting.

## 1. Introduction

Italy is the third largest producer of kiwifruit worldwide, after China, the native country of Actinidia, and New Zealand. In 2022, the production of these three countries amounted to about 80% of the global kiwifruit production [1]. The economic importance of this fruit is attributable to its taste and high nutritional and organoleptic quality. In terms of cultivars, green and yellow-fleshed kiwifruits dominate the global commercial market [2]. Among the green-fleshed cultivars, “Hayward” represents the most commercialised variety on the international market, covering about 90% [3]. This primacy is attributed to the size, taste, and storability of the fruit, which maintains good quality in terms of texture, flavour, and freedom from disorders during long postharvest storage [4]. In recent years, the green-fleshed kiwifruit production known as Actinidia chinensis var. deliciosa has been joined by yellow-fleshed cultivars (A. chinensis var. chinensis) [5]. The optimal harvest time for kiwifruit is typically when fruit softening has not yet started, obtaining and preserving optimal quality characteristics for postharvest storage and distribution [6,7]. Soluble solids content (SSC) and dry matter (DM) are used as important variables indicating harvest time, with values of about 6.2–6.5 °Brix for SSC and 15–20% for DM [8,9]. The European Commission Regulation (EC, 1221/2008) [10] states that kiwifruit must have a minimum content of 6.2 °Brix for the packing, import, and export stages and at least 9.5 °Brix for the subsequent marketing stage.

Since the harvest time affects the ripening quality of kiwifruit cultivars, in the last two decades many technologies have been developed and applied to reduce long, slow, and labour-intensive procedures to determine the exact kiwifruit harvest time. Among these, non-destructive methods, which are easy to operate, contactless, rapid, accurate, and non-polluting, have been applied for the quality assessment of products to predict their sensory and desired compositional traits objectively and consistently [11,12,13,14,15,16]. In kiwifruit, it has been demonstrated that it is possible to estimate glucose, DM, and SSC using near-infrared (NIR) spectroscopy [17,18]. Ma et al. (2021) [19] used a rotation NIR hyperspectral imaging camera to scan the whole kiwifruit surface to construct a predictive model with an R^2^ of about 0.74 and 0.64 for SSC and pH, respectively. The same parameters and the kiwifruit firmness were measured using a newly designed VIS–NIR spatially resolved spectroscopic system [20]. Mishra et al. (2023) [21] proposed a one-touch portable spectral image system in the range of 500–1000 nm that was used to predict SSC and DM of green and yellow-fleshed kiwifruit. A double-lightning imaging system, able to capture colour and UV-excited fluorescence images of green kiwifruit using specific wavelengths, was developed by Nie et al. (2020) [18]. In this trial, colour and fluorescence image features to predict SSC in a non-destructive way were used to determine the optimum harvest stage. Since visible imaging techniques are simpler than a spectroradiometer and use relatively low-cost equipment, an imaging system may be used to determine the fluorescence traits of the fruit related to quality change because fruit fluorescence emissions also occur in the visible range (e.g., fluorescence peak in the red region). The PLSR model used to predict the SSC achieved an accuracy of R^2^ of 0.89, starting from a combination of colour and textural features that were extracted from colour images and UV-fluorescence emission images generated by excitation at 370 nm. In this study, the capability of full-range (e.g., 400–2500 nm) portable hyperspectral measurements of surface reflectance on kiwifruit was evaluated in a controlled experiment on golden kiwifruits, with the objective of estimating the maturity index in a non-destructive manner. Spectral data were acquired on kiwifruit harvested from two farms, located in the same cultivation district during four development stages. These data were then used to calibrate and validate predictive models using different statistical tools. The predictive capability of the different selected models was assessed and compared with state-of-the-art computer vision-based technology, finally highlighting their potential to be scaled at the industrial level.

## 2. Materials and Methods

### 2.1. Plant Material

Fruit of *Actinidia chinensis* L. cv G3, also known as SunGold by Zespri^®^, were supplied by two farms (called F1 and F2 respectively) that are members of the cooperative company APOFRUIT Italia. Both farms are located in Scanzano Jonico (40°15′ N; 16° 42′ E; 50 m above sea level), in southern Italy. The Zespri^®^ quality harvesting protocol has been followed by APOFRUIT Italia for the kiwifruit orchards to ensure the correct taste and optimal shelf-life of the kiwifruits. Starting from the first week of September 2022, every 7–10 days, three and four harvest times (HT) were applied at farm one (F1) and farm two (F2), respectively, labelled as HT1, HT2, HT3, and HT4. After each HT, the fruit were immediately transported, in an air-conditioned van, to the postharvest laboratory of CNR-ISPA. The kiwifruit were subject to non-destructive analysis using a computer vision system and a portable spectroradiometer, followed by destructive qualitative analysis measuring firmness, titratable acidity, pH, SSC, dry matter, and soluble sugars. Ten replicates of six kiwifruits were used for each HT and each farm.

### 2.2. Non-Destructive Analysis

#### 2.2.1. Hyperspectral Methodology

Hyperspectral measurements were performed with a portable spectroradiometer (Spectral Evolution RS 5400, Haverhill, MA, USA) measuring radiance in the visible (VIS), near-infrared (NIR), and short-wave infrared (SWIR) spectrum between 350 and 2500 nm, using three different detectors, with a spectral resolution ranging between 2.5 and 5.8 nm based on the spectral region. The detectors and onboard electronics are tightly packaged in a metallic frame weighing less than 6 kg and compact in size (31.5 × 22.1 × 11.2 cm). Combined with the possibility of being battery-powered and operated via a tablet, the instrument is fully portable and field-rugged. Spectral data were acquired via a handheld fibre optics equipped with its own contact probe, inclusive of a calibrated light source. The instrument can therefore be used to acquire spectral information without a dedicated laboratory set-up and, in principle, can work directly in the field if needed. Three consecutive spectral measurements were taken on different spots along the equatorial circumference of the unpeeled whole fruit. Radiance data were converted to reflectance by taking reference radiances on a Spectralon^®^ high-reflectivity panel and computing the ratio between the two radiances. A reference radiance was taken at each replicate to account for any change in environmental or instrument operational conditions.

#### 2.2.2. Colour Parameters Acquired by the Computer Vision System

At each HT and for both farms (F1 and F2), the kiwifruit skin colour parameters were measured using a computer vision system (CVS). The CVS is equipped with an AP3200TPGE RGB digital camera (JAI Ltd., Yokohama, Japan), with a spatial resolution of 3.2 MP at 2 fps and a colour depth of 24 bit/pixel, and with a lens of 12 mm focal length and F1.8 (KOWA Lens mod. LM12NC3 1/2), providing a field of view of 35 × 30 cm. The camera was placed inside a HPB60D photo studio box (HAVOX, Vendôme, France), equipped with two LEDs, each consisting of 60 diodes, which provided the illumination used to capture images of the fruits. A colorimetric reference target (X-Rite ColorChecker Passport 24 patches) was located inside the box in the camera field of view. Images were processed using Matlab^®^ R2021b (MathWorks Inc., Natick, MA, USA). The raw image of the kiwifruit was isolated from the background to create a binary image, using the method described by Gonzalez et al. (2009) [22]. This algorithm removed unnecessary image borders and separated RGB (red, green, and blue) colour components from the raw images. The RGB data were converted into L*, a*, and b* colour spaces. Then, the Chroma and hue angle (°) were calculated from the primary L* a* b* readings, which were used in the following equations (Pathare et al., 2013) [23]

(1) $$Chroma=\sqrt{{a*}^{2}+{b*}^{2}}$$

(2) $$Hue\;angle={tan}^{-1}\left(\frac{b^{*}}{a^{*}}\right)$$

### 2.3. Destructive Analysis

#### 2.3.1. Firmness, Titratable Acidity, pH, Soluble Solids Content, and Dry Matter

The firmness of the kiwifruits was measured using the method initially proposed by Cefola et al. (2014) [24]. A texture analyser (ZwickiLine Z0.5, Zwick/Roell, Ulm, Germany) equipped with a 6 mm diameter probe with a flat end was used. The skin of each fruit was removed using a peeler, and measurements were taken at the equatorial region of the fruit. The force required to obtain a deformation of 2 mm was recorded for each fruit and the firmness was expressed as kg cm^−2^. About 100 g of kiwifruit for each replicate were homogenised (T 25 digital Ultra-Turrax-IKA, Staufen, Germany) to obtain the juice used to determine the titratable acidity (TA), pH, and SSC. Titratable acidity expressed as % of citric acid and pH were determined by a semi-automatic titrator/pH metre (PH-Burette 24 Crison Instrument, Barcelona, Spain) using 0.1 M NaOH to the final pH 8.1 for the titration. The SSC was measured using a digital refractometer (DBR35 XS Instruments, Carpi, Italy) and expressed in °Brix. To determine the dry matter (DM), chopped kiwifruit were dried in a forced ventilation oven (M700-TB, MPM Instruments, Bernareggio, Italy) at 65 °C for 24 h, until a constant mass was reached. The DM was calculated as the percentage ratio between the dry and fresh weight of the samples.

#### 2.3.2. Determination of Glucose and Fructose

Kiwifruits were freeze-dried until reaching a stable weight (about 46 h) using an Alpha 2–4 LSC plus freeze-dryer (Martin Christ Gefriertrocknungsanlagen GmbH, Osterode am Harz, Germany) with a vacuum pressure of 0.015 mbar and a condenser temperature of 60 °C. The freeze-dried fruit were ground at 500 µm by using a laboratory mill (Retsch Italia srl, Torre Boldone, Italy) to obtain a homogeneous powder. The glucose and fructose determination was made according to Montefusco et al. (2021) [25] by mixing triplicate aliquots (10 mg) of each sample with 70% ethanol (*v/v*) for 30 min. All the samples were centrifuged using a high-speed micro-centrifuge (SCILOGEX D3024, Rocky Hill, CT, USA) at 6000 g for 10 min and dried under nitrogen flux. The dried material was redissolved in 1 mL of water, filtered through a 0.45 μm syringe filter (Millipore Corporation, Billerica, MA, USA), and assayed by a 1100 Series HPLC system (Agilent Technologies Inc., Santa Clara, CA, USA) equipped with an Aminex HPX-87H column (300 × 7.8 mm) (Biorad, Hercules, CA, USA). The glucose and fructose contents were expressed as mg g ^− 1^ of fresh weight.

### 2.4. Statistical Analysis

The data obtained by the CVS and the destructive analysis were analysed by one-way analysis of variance (ANOVA) to evaluate the effects of the harvest time (HT) on the postharvest quality determinations (colour, TA, pH, SSC, DM, and firmness) of golden kiwifruits provided by each farm. The mean values (n = 10) were separated using the Least Significant Difference (LSD) test (*p* ≤ 0.05). The software used was Statgraphics (version 19-X64, Warrenton, VA, USA). Reflectance signatures of the kiwifruits (obtained using the hyperspectral methodology) were employed to evaluate the capabilities of different regression models to predict the SSC and the sugars in the fruit (glucose and fructose, both in the fresh and dried fruit). Three different approaches, as detailed in Table 1, were tested: (i) Partial Least-Squares Regression (PLS) via the PLS regression function; (ii) Support Vector Regression (SVR); and (iii) Gaussian process regression (GPR). All models were implemented through the Python 3.12 library scikit-learn [26]. A common data processing pipeline was adopted for all the models. To reduce the dimensionality of the dataset and avoid overfitting of redundant features [27], a feature selection approach was used to select the most informative wavelengths. Retained wavelengths were those that fell into the top 20th percentile of a univariate F-score using the Select Percentile function of the sklearn feature selection suite with F regression as a scoring function. F regression uses the F-score of a univariate cross correlation between the predictors and the response variable, and the feature selection is therefore repeated for each dependent variable (SSC, sugars, etc.). Input reflectance data standardisation was required only for the SVR model and was obtained by removing the mean and scaling to unit variance of each variable. No standardisation was required for the other models as the data themselves are well constrained in magnitude. After preprocessing, the incoming dataset was divided into a training section (67% of the total data) and a test section (33% of the total data). Then, a grid-search algorithm was applied for the optimisation of the hyperparameters of the selected models (Table 1). During this step, the model used the training dataset to test different sets of hyperparameters. For each set, the model was trained on the training section and the performance evaluated in terms of R^2^ on the test section. The resulting optimised hyperparameters were those that maximised R^2^. Dataset partitioning into training and test subsets was then randomly repeated 10 times using the selected optimal hyperparameters. This step was employed to assess the model’s robustness and to confirm that the parameter selection was not dependent on the specific training and test datasets that were used. The final performance of the model was evaluated as the mean R^2^ and mean square error (MSE) over the 10 trials.

## 3. Results and Discussion

### 3.1. Results on Colour Parameters Acquired by the Computer Vision System

The results of the one-way ANOVA, as reported in Table 2, highlight that the HT only affected the b* and Chroma values among the kiwifruit skin colour parameters measured by the CVS. In detail, a significant reduction in b* was recorded from HT1 to HT4 in F1 and F2, respectively. A similar reduction was also recorded for Chroma values in the last harvest time (HT4) (Table 2). Colour data obtained by CVS were correlated with the parameters affecting the maturity index and no significant relationships were found. Considering these results, the CVS was not able to assess the maturity index of kiwifruit.

### 3.2. Quality Parameters from Destructive Analysis

**Figure 1:**
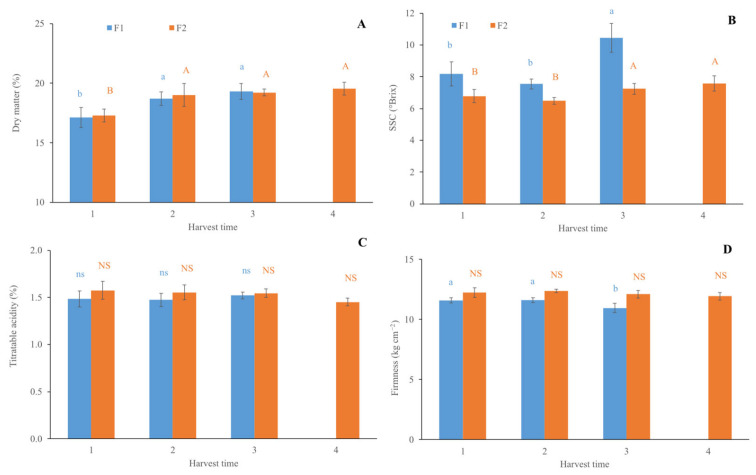
Quality parameters of golden kiwifruits assessed at each harvest time for farm one (F1) and farm two (F2): (**A**) dry matter; (**B**) soluble solids content; (**C**) titratable acidity; (**D**) firmness. Within the same harvest time, different letters indicate statistical differences (*p* ≤ 0.05) according to LSD test. ns or NS = not significant. Upper-case letters refer to F2 samples, while lower-case letters refer to F1 ones.

In Figure 1, the values of DM (A), SSC (B), TA (C), and firmness (D) of the kiwifruit measured at each HT in both farms (F1 and F2) are reported. Statistical differences evaluated by the LSD test were observed in all the postharvest quality traits among the HT, except for the TA. In detail, the DM of the fruit (Figure 1A) showed an increase of about 9% from HT1 to HT2 in both farms, remaining the same from HT2 to HT4.

**Figure 2:**
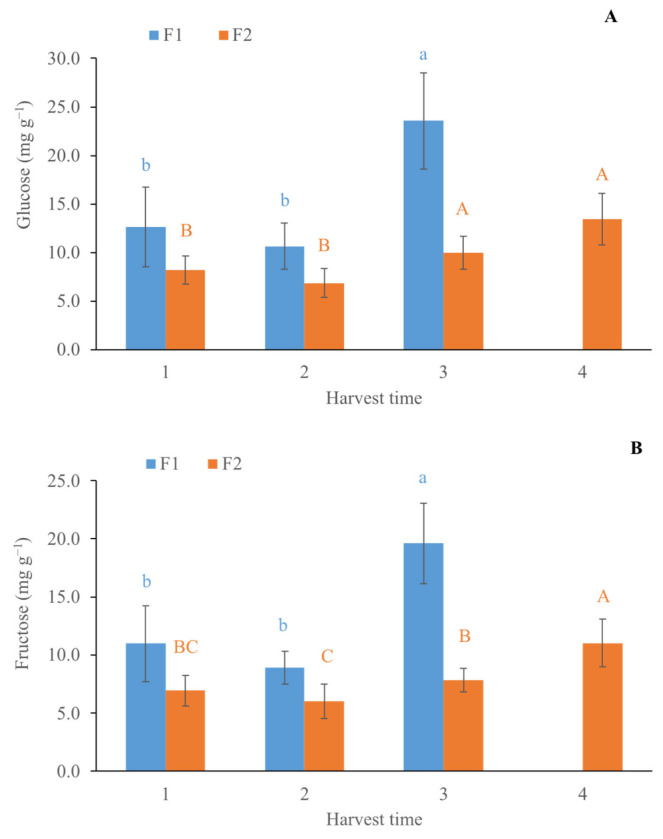
Content of glucose (**A**) and fructose (**B**) (expressed on fresh weight) of golden kiwifruit from farm one (F1) and farm two (F2) at each harvest time. Within the same harvest time, different letters indicate statistical differences (*p* ≤ 0.05) according to the LSD test. Upper-case letters refer to F2 samples, while lower-case letters refer to F1 ones.

Similar changes of DM are reported by Burdon et al. (2014) [4], who measured an increase in DM content of about 0.4% per week. As for the SSC (Figure 1B), a value of about 7.36 and 6.63 °Brix for F1 and F2, respectively, was preserved in HT1 and HT2, while a significant increase was recorded in HT3 (10.24 ± 0.9 and 7.44 ± 0.34 °Brix for F1and F2, respectively), without significant changes recorded in HT4 for F2 kiwifruit. Lim and Eom (2018) [9] reported a gradual increase in SSC of golden kiwifruit, reaching a value of 10.56 °Brix at the final harvest time (160 d after full bloom). No statistical differences were observed in TA among the HT for both farms (Figure 1C), while a significant reduction of about 5.3% in the firmness from HT1 to HT3 was only recorded in kiwifruit of farm F1 (Figure 1D). All the fruit experienced a gradual decrease in firmness during ripening, together with an increase in SSC and DM content to optimum values for harvest time [9]. Data obtained in our research confirmed that fruits from farm F1 were riper than those from F2. This result is also confirmed by the glucose and fructose content (Figure 2). The LSD test showed a significant increase (of about 44.0 and 11.4% for F1 and F2, respectively) at the HT3 due to the ripening of the fruit. In detail, the glucose content was higher (57.7%) in F1 than F2 at the HT3. A similar trend was exhibited by the fructose content (60.1% higher in F1 at HT3). The data obtained by destructive analysis indicate that the kiwifruit of the two farms had a different level of maturity. Specifically, in the first HT, the fruit from F1 was riper than that from F2; for this reason, four HTs were necessary for F2 and only three for F1.

### 3.3. Prediction of Soluble Solids Content and Sugars by Hyperspectral Analysis

**Figure 3:**
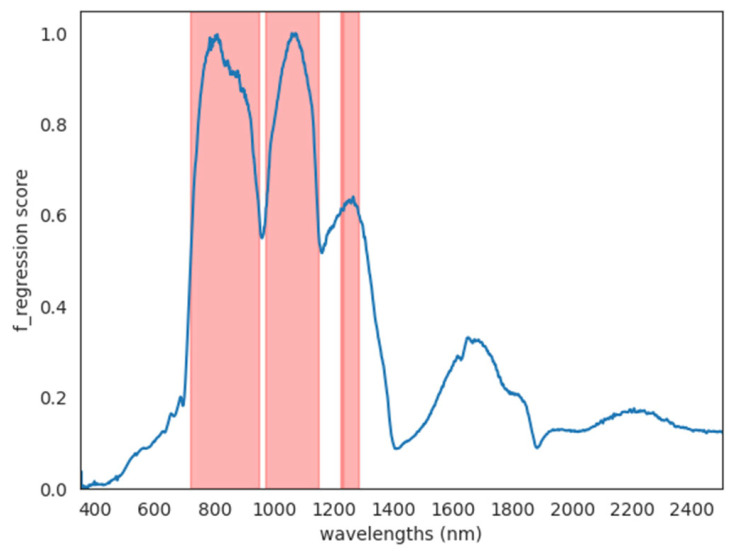
Dimensionality reduction results for feature selection algorithm. The feature selection algorithm, utilising F regression as a scoring function, consistently selected wavelengths (highlighted in red) in the range of 719 to 1280 nm across all iterations, each corresponding to a different response variable. Notably, there were gaps in the selected wavelengths in the regions between 950 and 971 nm, 1146 and 1223 nm, and 1227 and 1232 nm. These gaps indicate specific wavelength ranges that were excluded during the selection process.

The dimensionality reduction algorithm, which considered all the iterations of the feature selection step (one per each response variable), always selected wavelengths between 719 and 1280 nm, with some gaps (between 950 and 971 nm, between 1146 and 1223 nm, and between 1227 and 1232 nm), therefore selecting most of the NIR region and the initial part of the SWIR region. No wavelengths in the VIS (350–700 nm) or in the further region of the SWIR (above 1280 nm) were selected (Figure 3).

Given the wavelengths chosen by the feature selection step, the results of the modelling step are summarised in Table 3.

The results show that the models perform differently depending on the response variable. On average, PLS and GPR are confirmed as good models for SSC and sugars with comparable performances. While the GPR model demonstrated superior performance for fructose prediction, PLS regression yielded optimal predictive accuracy for SSC and glucose content. SVR, on the other hand, shows a worse performance on both sugars and soluble solids content than the other two models, with the exception only of fructose in the fresh fruit, where the average R^2^ of SVR is 0.54 and the average R^2^ of PLS is 0.58. It must be considered, though, that these are average R^2^ coming from 10 iterations of the models, and the standard deviation is often similar for PLS, SVR, and GPR. In terms of MSE, the overall GPR showed lower average errors compared to the other two models. Feature selection is a crucial part of the modelling process, given that it determines the input dataset for the various models. In this case, the feature selection chose most of the NIR spectrum, a small part of the SWIR spectrum, and completely excluded both the visible range and the SWIR above 1280 nm. This is in accordance with the experiments of Mishra et al. (2023) [21], which applied VIS–NIR spectroscopy and PLS to predict DM matter and SSC on both green and gold kiwifruits. The authors tested the PLS with two spectral ranges, one including the VIS + NIR (500–1000 nm) and one the NIR only (688–1000 nm) on green kiwifruits and then applied the output models to gold kiwifruits. The authors concluded that for the gold kiwifruit model, performances were similar for VIS + NIR and NIR only, thus suggesting that for the prediction of DM and SSCs in gold kiwifruit, the information contained in the VIS portion of the spectrum was not particularly relevant.

The relevant spectral regions highlighted by the feature selection and not containing the visible bands confirm the results obtained here for the CVS, which is based on RGB imaging and was found to be incapable of estimating fruit maturity. A similar selection of relevant spectral features located above 750 nm when predicting SSC in kiwifruit with hyperspectral data was also found by Xu et al. (2024) [28] using a near-infrared (VIS–NIR at 400–900 nm) system. To the best of our knowledge, this is the first study in which a fully portable spectroradiometer measuring surface reflectance in the full VIS–NIR-SWIR range has been used on kiwifruits.

Recently, the inclusion of the SWIR spectral region when attempting to explain the biophysical properties of ecosystems has been found to be important (Heidarian Dehkordi et al., 2024) [29], suggesting that previous studies mostly focusing of the NIR region were more limited by the technological requirements to measure SWIR reflectance with sufficient accuracy, rather than by the lack of significant relations between plant or fruits traits and SWIR reflectance.

It is worth noting that, beside the standardisation requirement for the SVR model, no other preprocessing was carried out on the reflectance spectra. This choice was made following Mishra et al. (2021, 2023) [21,30], who found better results with raw reflectance data. Discussing model performances is not straightforward due to the variety of different metrics employed in the literature, which make direct comparisons challenging. Guo et al. (2016) [31] used hyperspectral NIR data with and without feature selection to predict SSC in two kiwi varieties (“Xixuan” and “Huayou”), with PLS and the least-square support vector machine (LS-SVM). The authors obtained the root mean square error (RMSE) of prediction, which varied between 0.91 and 2.36 for PLS and between 0.59 and 1.15 for LS-SVM. In this study, taking the square root of the MSE ± its standard deviation, we obtained a RMSE of 0.69–1.06 for PLS and 0.73–1.36 for SVR. Guo et al. (2016) [31] reported a better performance for LS-SVM than for PLS, while in our study the minimum RMSE for SSC was observed in PLS (0.69 vs. 0.73 for SVR) and SVR exhibited a higher RMSE compared with PLS (1.36 vs. 1.06). These differences in performance between PLS and support vector models could be due to different factors. First, Guo et al. 2016 [31] used a spectral range between 865.11 and 1711.71 nm; this does not consider the bands around 700 nm, which were important in the feature selection step of our study and that of Mishra et al. (2023) [21]. Second, the support vector approach used was different given than in our study, a support vector regression with a linear kernel was employed versus a least-square approach as in [31]. SVR and LS-SVM are both regression techniques based on the principles of SVM, but they differ in their optimisation objectives and computational approaches. SVR focuses on maximising the margin around the regression line, while LS-SVM reformulates the problem to minimise the least squares loss. Third, kiwifruit varieties were also different, yielding potentially different spectral signatures and model performances. Hu et al. (2017) [32] also used VIS–NIR hyperspectral imaging to predict sugar content in Hayward kiwifruits, both treated and untreated with 1-methylcyclopropene. The authors analysed both whole fruits and slices and employed both PLS and LS-SVM with feature extraction. When considering the intact fruit, the whole spectral range, and both the treated and untreated fruit, Hu et al. (2017) [32] found, for glucose and PLS, an R^2^ ranging between 0.62 and 0.63 and RMSEs around 4.8 mg g^−1^.

In this paper, we obtained an R^2^ between 0.42 and 0.74 and a lower RMSE (2.89–3.85 mg g^−1^). LS-SVM, on the other hand, obtained an R^2^ and RMSE for glucose of 0.63 and 4.75–4.78 mg g^−1^ respectively, which are within the range of what was obtained here by SVR (R^2^ 0.41–0.67, RMSE 3.92–5.85 mg g^−1^). For fructose, LS-SVM yielded R^2^ (0.52-0.62) which are similar to the ones obtained in the present work (0.35–0.61 for SVR), but a higher RMSE (3.45–4.18 mg g^−1^ for LS-SVM vs. 2.82–4.07 mg g^−1^ for SVR). Again, differences in the modelling approach (LS-SVM vs. SVR) might explain these differences in outcomes. Unfortunately, it was not possible to find applications of GPR to kiwifruit in the literature, but [33] both PLS and GPR had been used to predict SSC on fresh cherry fruit using hyperspectral imaging. In that case, GPR (R^2^ 0.88, RMSE 0.43) yielded better results than PLS (R^2^ 0.71, RMSE 0.64) for SSC. In our case, the performance of the two models in estimating SSC was quite similar, but it is challenging to make direct comparisons due to the important difference between the two fruits.

One advantage of a GPR-based model is its ability to achieve precise optimisation, similar to other kernel methods, which enables a balanced trade-off between fitting the data and smoothing. Moreover, GPR models are particularly suited to handling small datasets due to their effective smoothing capabilities and computational efficiency [34].

## 4. Conclusions

The use of a full-range spectroradiometer coupled with selected predictive models was proposed as a rapid, contactless, and non-destructive method, as a practical tool for kiwifruit producers to estimate the maturity index and optimise the harvesting time. To the best of our knowledge, this is the first study in which a fully portable spectroradiometer measuring surface reflectance until the full SWIR range has been used on kiwifruits. Feature selection showed contributions of SWIR wavelengths below 1280 nm, and future research is therefore warranted on the importance of the SWIR range of the spectrum for the determination of fruit characteristics. It is also worth noting that the portability of the instrument makes it possible to replicate these measurements anywhere without the need for dedicated conveyor belts, stepper motors, or other laboratory equipment to position the fruit under the sensor.

Although the spectroradiometer used in this trial is an instrument for research applications, with a relatively high cost, the results can drive the industrial development of simplified and optimised devices that measure only in the sensitive spectral bands, with a reduced weight, size, and cost. Such devices could also be used directly in the field provided that direct contact between the fruit and the sensing probe is provided.

## Figures and Tables

**Table 1:** Hyperparameters of the models, including their corresponding model names, abbreviations, and the minimum and maximum limits of the search ranges for optimisation.

Model	Hyperparameter Name	Hyperparameter Description	Range Min.	Range Max
PLS	NCOMPS	Number of components retained by PLS	1	40
SVR	EPS	Defines the distance from the predicted to the actual value (i.e., “tube” width) for which no penalty is applied in the training loss function	1 × 10^−40^	1 × 10^3^
SVR	C	Regularisation strength	1 × 10^−3^	1 × 10^5^
GPR	LSCALE	Length scale of RBF kernel	1 × 10^−4^	1 × 10^4^
GPR	ALPHA	Noise level	1 × 10^−20^	2

**Table 2:** Colour parameters of kiwifruit assessed by the computer vision system at each farm (1 and 2) and harvest time (HT1, HT2, HT3, HT4).

Colour Parameters	Farm 1							Farm 2								
	HT1		HT2		HT3		*p*-Value	HT1		HT2		HT3		HT4		*p*-Value
L*	17.98		17.38		17.40		ns	17.44		16.60		16.39		17.29		ns
a*	2.87		2.83		3.09		ns	3.60		3.42		3.44		3.16		ns
b*	13.67	a	13.10	ab	12.79	b	*	13.38	a	12.45	b	12.28	b	12.21	b	**
Chroma	13.99	a	13.42	ab	13.16	b	*	13.87	a	12.92	b	12.76	b	12.68	b	**
hue angle	78.13		77.77		76.34		ns	74.95		74.57		74.29		74.84		ns

Mean values followed by different letters within columns are significantly different (*p* ≤ 0.05). ns: not significant, *p* ≤ 0.05 (*), *p* ≤ 0.01 (**).

**Table 3:** Summary of model performance for Partial Least Squares (PLS), Support Vector Regression (SVR), and Gaussian Process Regression (GPR), including hyperparameters. Reported metrics include the average (AVG) and standard deviation (STD) of the coefficient of determination (R^2^) and mean squared error (MSE) across validation folds.

**Prediction of Soluble Solids Content (°Brix)**							
model	stage	min	max	mean	std	R^2^ avg	MSE avg
PLS	test	6.22	11.39	7.75	1.32	0.60 ± 0.16	0.75 ± 0.26
PLS	train	6.03	11.58	7.75	1.31	0.77 ± 0.06	0.39 ± 0.08
SVR	test	6.22	11.39	7.75	1.32	0.55 ± 0.13	0.97 ± 0.27
SVR	train	6.03	11.58	7.75	1.31	0.75 ± 0.08	0.49 ± 0.15
GPR	test	6.22	11.39	7.75	1.32	0.6 ± 0.12	0.89 ± 0.16
GPR	train	6.03	11.58	7.75	1.31	0.99 ± 0.00	0.0 ± 0.0
**Prediction of Glucose in fresh kiwifruit** **(mg g^−1^)**							
model	stage	min	max	mean	std	R^2^ avg	MSE avg
PLS	test	5.89	29.56	12.23	5.88	0.58 ± 0.16	17.38 ± 5.34
PLS	train	4.46	30.78	12.17	5.69	0.77 ± 0.08	7.31 ± 1.97
SVR	test	5.89	29.56	12.23	5.88	0.54 ± 0.13	19.48 ± 4.09
SVR	train	4.46	30.78	12.17	5.69	0.78 ± 0.09	7.66 ± 2.35
GPR	test	5.89	29.56	12.23	5.88	0.55 ± 0.12	18.22 ± 3.72
GPR	train	4.46	30.78	12.17	5.69	0.99 ± 0.00	0.37 ± 0.06
**Prediction of Fructose in fresh kiwifruit (mg g^−1^)**							
model	stage	min	max	mean	std	R^2^ avg	MSE avg
PLS	test	4.70	24.24	10.29	4.87	0.58 ± 0.16	11.59 ± 3.22
PLS	train	3.72	24.37	10.13	4.55	0.76 ± 0.08	4.92 ± 1.03
SVR	test	4.70	24.24	10.29	4.87	0.48 ± 0.13	14.68 ± 2.80
SVR	train	3.72	24.37	10.13	4.55	0.76 ± 0.09	5.55 ± 1.94
GPR	test	4.70	24.24	10.29	4.87	0.59 ± 0.12	11.29 ± 1.58
GPR	train	3.72	24.37	10.13	4.55	0.99 ± 0.00	0.27 ± 0.03

Hyperparameters include the number of components (NCOMPS), epsilon (EPS), regularisation parameter (C), length scale (LSCALE), and regularisation strength (ALPHA). Specifically, NCOMPS was set to 6 for fresh and glucose, and 7 for Soluble Solid Content. For SVR, EPS and C were 1.5 and 1000 for Glucose and Fructose and 2 and 100,000 for Soluble Solid Content. For GPR, LSCALE and ALPHA were 2 and 1 × 10^−5^ for Glucose and Fructose, and 10 and 1 × 10^−10^ for Soluble Solid Content.

## Data Availability

The original contributions presented in the study are included in the article, further inquiries can be directed to the corresponding author.
